# Intensity Weighted Subtraction Microscopy Approach for Image Contrast and Resolution Enhancement

**DOI:** 10.1038/srep25816

**Published:** 2016-05-12

**Authors:** Kseniya Korobchevskaya, Chiara Peres, Zhibin Li, Alexei Antipov, Colin J. R. Sheppard, Alberto Diaspro, Paolo Bianchini

**Affiliations:** 1 Nanoscopy, Nanophysics, Istituto Italiano di Tecnologia, Via Morego 30, 16163 Genova, Italy; 2 Advanced Robotics, Istituto Italiano di Tecnologia, Via Morego 30, 16163 Genova, Italy; 3 Plasmachem GmbH, Rudower Chaussee, 29, D-12489, Berlin, Germany

## Abstract

We propose and demonstrate a novel subtraction microscopy algorithm, exploiting fluorescence emission difference or switching laser mode and their derivatives for image enhancement. The key novelty of the proposed approach lies in the weighted subtraction coefficient, adjusted pixel-by-pixel with respect to the intensity distributions of initial images. This method produces significant resolution enhancement and minimizes image distortions. Our theoretical and experimental studies demonstrate that this approach can be applied to any optical microscopy techniques, including label free and non-linear methods, where common super-resolution techniques cannot be used.

In the last two decades, tremendous progress has been made in optical microscopy techniques^1^,^2^,^3^. Today, sub-diffraction resolution can be achieved by fluorescence microscopy, thanks to newly developed approaches such as stochastic optical reconstruction microscopy (STORM), photoactivated localization microscopy (PALM), stimulated emission depletion microscopy (STED), reversible saturable optical fluorescence transitions (RESOLFT) together with the further improved time-gated STED (g-STED), saturated structured illumination microscopy (SSIM), and other variations on the super resolved fluorescence microscopy theme^4^,^5^,^6^,^7^,^8^. However, all these methods require expensive and complex equipment, which is difficult to be assembled by non-specialists. Moreover, these techniques cannot be applied to some types of label-free imaging methods, such as second-harmonic generation (SHG) microscopy or reflection confocal microscopy.

Recently, as an alternative solution, an image subtraction approach was proposed for the contrast and resolution enhancement in laser scanning microscopy techniques, known as switching laser mode (SLAM) or fluorescence emission difference (FED)^9^,^10^. Both techniques are based on the concept that two images are consequently taken with Gaussian and doughnut shaped excitation beams, and afterwards the images are subtracted from each other with an appropriate normalizing coefficient.

The concept of subtraction is based on the early photographic principle of unsharp masking, in which a blurred image is subtracted from an in-focus image. Such an approach has also been employed in image processing, and is similar to edge enhancement by convolving with a kernel. A conventional image can also be subtracted from a confocal image^11^, or two confocal images recorded with different pinhole sizes can be processed^12^,^13^. However, in these cases the intensity at the centre of the point spread function (PSF) is greatly reduced by subtraction, whereas using a doughnut beam with a dark centre is greatly advantageous as the intensity at the centre of the PSF is unchanged. Ideally we want to subtract away the outer parts of the central lobe of the PSF, while leaving the centre of the central lobe, and the outer regions unchanged, thus specifying the ideal form for the doughnut beam.

Leray and Mertz proposed a similar subtractive method for two-photon excitation (2PE) fluorescence^14^, but in their case the justification was to remove background in order to increase penetration depth. The focal modulation microscopy technique also has some similarities^15^, and the resolution enhancement properties have been investigated^16^.

Subtraction is usually regarded as not optimal, as the signal is reduced while the noise is increased. However, it has been shown that for confocal fluorescence with a finite sized pinhole, the optical transfer function can actually go negative, so that subtraction can actually increase signal for particular bands of spatial frequencies^17^. Defocus or aberrations can also result in negative values for the transfer function^18^, and polarization can also have a similar effect^19^.

Deconvolution is another image post processing approach which can provide good resolution enhancement up to factor of two in some cases^20^. However, the resolution obtained by the deconvolution process is strictly dependent on quality of the prior information introduced in the algorithm, such as the sample morphological properties and the PSF. The lack of prior information may result in artefacts in the image, and thus resolution improvement could be very moderate or even absent. For this reason, there is a necessity of studying the image processing techniques, such as subtraction, which do not require prior information.

Therefore, the main novelty of FED and SLAM techniques is in using a combination of Gaussian and doughnut beams at the apparatus level, allowing subtraction to be performed without significant intensity loss. The resulting resolution enhancement of these techniques has been demonstrated to exhibit an improvement of up to a factor of two compared with the original confocal image^9^,^21^. Furthermore, it has been shown, that subtraction can be successfully applied for different imaging modes including label-free microscopy methods, such as, for instance, SHG and coherent anti-Stokes Raman-scattering (CARS) microscopies^22^,^23^,^24^.

Though the subtraction methods cannot provide resolution comparable with the aforementioned super resolution techniques, they are low cost, fairly simple to use and implement, and the required beam paths can be easily assembled in the laboratory. Compared to the super-resolution techniques that exploit nonlinear processes, subtraction microscopy is advantageous for *in vivo* imaging because it requires a lower laser power density on to the sample as well as shorter pixel dwell time, and hence minimizes photo-damage on living specimens. Additionally, this approach can be applied to any laser scanning microscopy method, and is particularly useful for label-free imaging, where the common super resolution methods cannot be used.

Unfortunately, the weak point of the subtraction techniques is the balancing of the initial images intensities, because the direct subtraction of normalized data sets often generates areas with negative intensity values, which are a potential source of data loss. For that reason, in both FED and SLAM microscopies, an empirically defined intensity rescaling coefficient for the image acquired by means of the doughnut shaped beam is introduced. In fact, Wang *et al.* showed that the optimum value for this coefficient is strictly dependent on sample type and imaging mode. It means that the coefficient needs to be adjusted with respect to all the parameters, including sample size and shape, numerical aperture of the objective lens, and even the polarization of the doughnut beam^25^. So far, subtraction images were obtained by manual selection of the most appropriate coefficient. Such an approach is not only time consuming, but may also introduce image distortion at times, which would be difficult to notice with crowded and tangled samples.

To resolve this problem, we propose a novel and universal algorithm for image subtraction, which we call intensity-weighted subtraction (IWS). The subtraction coefficient is chosen and assigned pixel-by-pixel with respect to the intensity difference between the original images. Our suggested approach not only enhances image contrast and resolution but also avoids over-subtraction and image distortions. To validate the IWS concept, we conducted investigations on different imaging techniques such as confocal reflection and fluorescence, two-photon excitation fluorescence, and second harmonic generation. We compared the results obtained from samples with different structural complexities, ranging from nanoparticles up to biological specimens, where structures such as cell microtubules and collagen fibres were studied. Overall, we were able to achieve up to 30% improvement in resolution with respect to the confocal image.

## Materials and Methods

### Reflection and single-photon fluorescence

For reflection and single-photon excitation fluorescence measurements we used a Leica TCS SP5 STED-CW (Leica-Microsystems, Mannheim, Germany) confocal microscope. The microscope is equipped with a pulsed white light laser (WLL), with which up to eight laser lines can be selected simultaneously in the spectral range 470–670 nm. The doughnut beam is at a fixed wavelength, i.e. 592 nm, realized by a high power CW laser. We acquired confocal reflection images at 592 nm, alternating by Gaussian and doughnut beams line by line in order to avoid non-uniform bleaching and to minimize the effect of small sample drifts, which may adversely affect the quality of the subtraction process. All the images were acquired using a pixel size of 50 nm with a pixel dwell time of 4,9 μs and 32 line averages. The laser power was properly chosen to be out of the saturation regime.

### Two-photon and SHG setup

The home-built experimental setup (Supplementary Fig. S1) is based on a Chameleon Ultra II (Coherent Inc., Santa Clara, CA, USA) femtosecond laser source with the following parameters: 80 MHz, 200 fs at 860 nm. The output beam is split into two parts with a 70/30 beam splitter (BS), both of which are collimated. The reflected portion propagates via a vortex phase plate in order to generate a doughnut shaped beam. Subsequently, both parts are recombined using a polarizing beam splitter, and circularly polarized with a combination of half and quarter wave plates. The laser lines are coupled with a commercial Nikon C2 confocal scan head (Nikon Instruments, Tokyo, Japan) and focused on the sample with an oil immersion objective, 100x NA 1.32. The backward signal is collected with a focusing objective and passes through the dichroic mirror, in order to reject the excitation wavelength, and then, in case of SHG, is guided to a non- descanned photomultiplier tube (PMT1 in Supplementary Fig. S1), while for two-photon fluorescence microscopy the signal is collected by the internal PMT after a pinhole that we kept open. The forward signal is collected with an air objective (10x) that is coupled with a PMT (PMT2 in Supplementary Fig. S1). The images are acquired by Nis-Elements software (Nikon Instruments, Tokyo, Japan).

### 3D confocal reflection imaging setup

The setup used to perform 3D confocal reflection imaging is a custom made microscope^5^ based on a pulsed Fianium (Fianium Ltd, Southampton, UK) 20 MHz high-power laser, endowed with a broadband super-continuum output. The Gaussian beam is selected from the super-continuum output by means of an AOTF and the wavelength is set at 710 nm. In order to obtain a bottle beam, we used the 710 nm fixed output and we placed a home made phase plate to introduce a phase delay of π in the centre that creates the z-doughnut for the increase in resolution in the axial direction. The Gaussian and the bottle beams are then combined by an appropriate dichroic mirror, scanned using two galvanometer mirrors and focused on the sample with a Leica 100× 1.25NA oil immersion objective. The reflected light is collected backwards through a fixed pinhole (1 Airy unit size) by an avalanche photodiode module (SPCM-AQRH-13-FC, Perkin Elmer, Vaudreuil, Québec, Canada).

### Sample preparation

The silver nano-wires (Plasmachem GmbH, Berlin, Germany) sample was prepared starting from a dispersed solution in methanol. After sonication, we drop casted it on piranha treated cover-glass slides^26^.

To image microtubules, we labelled HeLa cells as follows. The cultured cells were fixed in 4% PFA and then incubated with a monoclonal mouse anti-β-tubulin antibody (Sigma Aldrich, St. Louis, MO, USA) diluted in 3% BSA 0.1% Triton/PBS (1:1000) for 1 h at room temperature. After that we incubated them with ATTO-590 (ATTO-Tec GmbH, Siegen, Germany) conjugated goat anti-mouse antibody. The full procedure is described elsewhere^27^. The coverslips were successively immersed in solutions with increasing TDE concentrations (10%, 25%, 50% and 97%) for about 5–10 min each to optimize the red fluorophore performances^28^. The sample was sealed with nail polish.

The collagen sample was obtained from Bovine lateral meniscus. For sample preparation the meniscus was cleaned from other connective tissue and cut in cuboids with 1 cm side. After that, the cubes were fixed with 4% formalin and cryo-sectioned into 10 μm slices. Each slice was mounted on a cover glass and sealed using a 97% TDE solution.

## Results and Discussion

As we mentioned already, the key problem of subtraction microscopy is the selection of an appropriate subtraction coefficient in order to avoid over-subtractions and image artefacts. For SLAM and FED microscopy, the resulting image is obtained by intensity subtraction, as follow:


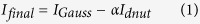


where *I*_*final*_, *I*_*Gauss*_ and *I*_*dnut*_ are the normalized intensity distributions of the resulting image, and the Gaussian and doughnut measurements, respectively. Clearly, the direct subtraction *I*_*Gauss*_ − *I*_*dnut*_ would inevitably create negative values, so it is necessary to introduce the subtraction coefficient *α* (equation (1)), in order to minimize this effect.

Despite the fact that some efforts were made to find the optimal subtraction coefficient analytically^21^,^25^, to date most researchers rely on an experimentally defined *α*. By analyzing simulated images of objects with different sizes and shapes, Wang *et al.* concluded that the subtraction factor should be set in a way that the peak intensity value of the final image is not smaller than 60% of its initial value^25^. For most cases the optimal value of *α* was shown to be about 0.5–0.6, while the negative values of intensity, which inevitably appear as a result of image subtraction, are usually set to zero^21^,^25^. Such an operation is indeed not critical for sparse samples, where the contrast structures are significantly smaller than the wavelength of the imaging system, and the distance between the closest object pairs is greater than λ/2. For instance, it has been shown that for sparse particles distribution the coefficient could be chosen between 0.7–1.0^21^.

The drawback of such an approach becomes evident for imaging dense objects of dispersed size and shape, where over-subtraction in overlapped areas might result in significant data loss and image distortion. For this reason, in all the aforementioned studies, the authors recommended application of a constant subtraction coefficient within the range from 0.5 up to 0.7^9^,^10^,^21^. However, in some cases, even low values of *α* may introduce image artefacts. This is well illustrated by the example shown in Supplementary Fig. S2, where in the crowded region over-subtraction starts to appear even for α = 0.5, and becomes essentially critical at α = 0.7. This observation is consistent with the study of Wang *et al.*^25^, where the authors show that for relatively big objects with dimensions of 0.3 λ over-subtraction occurs already by α = 0.6.

To address this problem, we propose the intensity-weighted subtraction (IWS) method, where the subtraction coefficient, α, is particularly assigned at each pixel based on the intensity distribution of the initial images. In general, it is desirable to avoid negative intensities in the subtracted image. Therefore, one way to achieve this is to use a large subtraction coefficient when the difference between intensity distributions of the Gaussian and doughnut images is large, and a small coefficient when the difference is small. Thus in areas with strong intensity fluctuations, a matrix of variable subtraction coefficients could outperform a constant one by adjusting the level of subtraction depending on the local differences.

**Figure 1:**
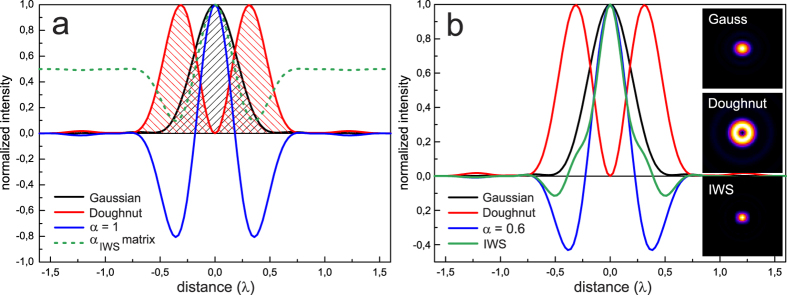
Intensity profile plots of simulated PSFs: black and red lines are the Gaussian and doughnut PSFs, respectively; (**a**) shows their direct subtraction (α = 1, blue curve) and weighted subtraction coefficient distribution α_IWS_ (green), (**b**) shows the resulting PSFs after IWS subtraction (green) compared with a constant value of α = 0.6 (blue). Inset demonstrates images of corresponding PSFs.

In order to illustrate such a concept, we simulated the profiles of a Gaussian (Fig. 1(a) black line) and doughnut (Fig. 1(a) red line) PSFs, and we plot their direct subtraction (Fig. 1(a) blue line). The PSF outlines were obtained using the calculation routine for the circular polarized Gaussian and vortex phase plate generated doughnut beams^29^. Clearly, maximizing the α coefficient within the common area of both PSFs, and minimizing it for the remaining part of doughnut PSF can lead to the best subtraction performance.

Hence we designed an approach, which allows performing an adaptive and variable subtraction, by taking into account the distinctive features in different regions of the acquired images. The weighted subtraction coefficient is obtained as follows:


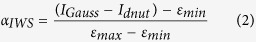


where I_Gauss_ and I_dnut_ are the intensity distributions of Gaussian and doughnut images respectively, *ε*_*min*_ *and ε*_*max*_are the boundary limits of the intensity values of the resulting image, which in this case we set between −1 and 1, as the theoretical minimum and maximum values for the normalized initial images.

If *a*_*11*_, *…a*_*nm*_ and *b*_*11*_, *…b*_*nm*_ are the coefficients of *I*_*Gauss*_ and *I*_*dnut*,_correspondingly, the 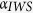 is calculated for each pixel as follows,


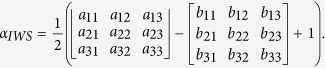


So we obtain the matrix of the subtraction coefficients which are then applied in equation 1. Writing *c*_*11*_, .. *c*_*nm*_ as the coefficients of *I*_*final*_, the operation can be written as follows:


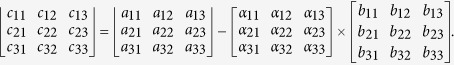


We plot the resulting α_IWS_ for 2D simulated PSFs in Fig. 1, as the green dashed curve. The weighting coefficient takes its highest values within the Gaussian PSF where over-subtraction is highly unlikely, and the lowest values are assigned to the regions where the *PSF*_*Gauss*_ − *PSF*_*dnut*_ becomes negative and over subtraction is then unavoidable.

It is worth stressing that direct subtraction of normalized PSFs (blue line) although it results in an improved full width at half-maximum (FWHM), generates undesired and excessive negative side lobes.

After obtaining the matrix of α_IWS_ values, we apply this instead of the constant α in equation (1). Figure 1(b) presents the resulting PSF_IWS_ compared with PSF_α=0.6_ shown as green and blue curves, respectively. As we can see from the picture, the IWS method decreases the undesired negative side lobes, which introduce image distortions in the conventional approach, while the resulting FWHM remains comparable with the case of constant subtraction. For the simulated diffraction limited PSFs, the estimated FWHM of the Gaussian PSF is about 0.44λ, while the resulting IWS spot size is reduced to 0.31λ, which corresponds to 30% resolution improvement. The slight broadening of the lower part of PSF_IWS_ can be neglected, because, as we will show later from the experimental data, it does not introduce artefacts and generally does not affect the resolution enhancement either, but in some particular cases it may slightly decrease the enhancement performance. To our understanding, it is of minor disadvantage compared to over subtraction, because the latter causes information loss, which may result in the misinterpretation of data. The presented simulations were performed for a doughnut beam geometry generated by a vortex phase plate, but any other engineered PSF featuring a zero in the centre, e.g. a Laguerre-Gauss beam, lead to similar or even better results when the hole diameter of the doughnut beam is narrower than the presented one.

In order to study the performance of the IWS method, we compared confocal reflection images of silver nano-wires (Ag NW) sample^26^, where several wires were crossing each other, with simulated images, which roughly resembled the original object geometry. The simulated images were obtained by convolving the phantom prototype with 2D simulation of the Gaussian and vortex doughnut PSFs (for the details, see Supplementary information, Fig. S3).

**Figure 2:**
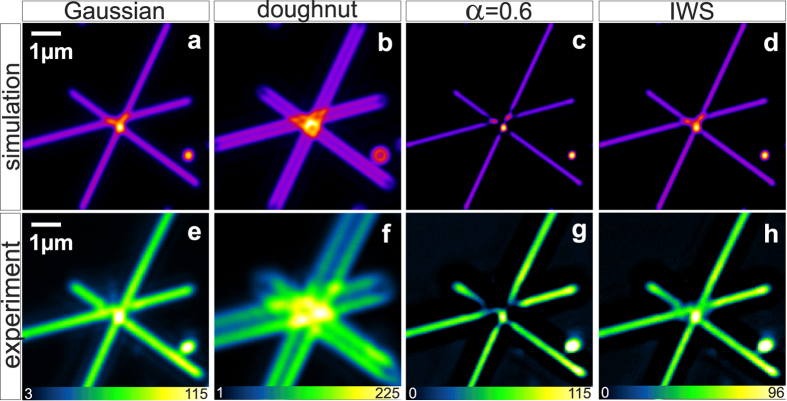
Simulated (top row) and experimental (bottom row) images of Ag NW in reflection at 592 nm, where (**a**,**e**) and (**b**,**f**) are confocal images resulting from Gaussian and doughnut beams, respectively. Applying constant subtraction method, with α = 0.6, to the pairs (**a**,**b**) and (**e**,**f**) we obtain (**c**,**g**) respectively, while applying IWS method to the same image pairs we obtain (**d**,**h**).

Confocal reflection images of Ag NW are shown in Fig. 2(e–h). The sample is a sparse particles distribution on a cover slip, obtained by the drop casting method. The average diameter of the NW is about 50 nm, while the length varies in a broad range. The confocal system was a Leica TCS STED-CW SP5 microscope, the illumination wavelength was 592 nm, the objective was a 100x NA 1.4 oil immersion, and the pinhole size was 0.5 Airy unit.

As we can see from Fig. 2(c,g), subtraction with a constant coefficient α = 0.6 produces data loss due to over-subtraction in the region where rods are overlapped. This occurs for both simulated and experimental data sets. At the same time using IWS subtraction mode helps to preserve the original features, without affecting the resolution improvement.

**Figure 3:**
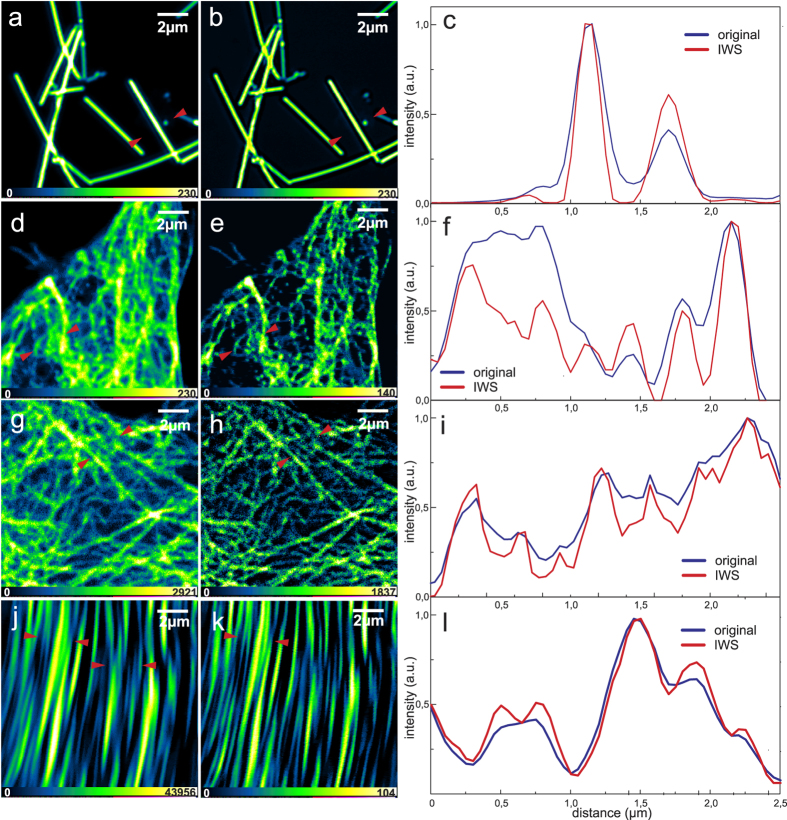
Left and middle columns show the original and IWS images respectively, the right column shows the intensity profiles along the line marked by the red arrows. In particular, (**a–c**) show reflection images of Ag NW; (**d–f**) are 1PE and (**g**,**h**) 2PE images of HeLa cell microtubules; (**j–l**) are SHG images of collagen fibres.

To further justify the concept’s performance, we applied the IWS method for various linear and non-linear modalities, presented in Fig. 3, such as confocal reflectance (a,b) and single-photon fluorescence (d,e); 2PE (g,h), and SHG in forward acquisition (j,k). The left column (a,d,g,j) shows diffraction limited microscopy images and the middle one (b,e,h,k) is the resulting intensity distributions after the IW subtraction. In all experiments, the power of Gaussian and doughnut beams were adjusted in a way that intensity values of corresponding images were similar.

The significant difference between confocal and IWS images is visually noticeable. Overall, the image sharpness and contrast became higher after subtraction. From intensity profiles (right column), we can see that the valleys between peaks became more pronounced. Some small features that could not be resolved by confocal imaging are successfully resolved after weighted subtraction. For instance, the two collagen fibres imaged at 860 nm (Fig. 3(l)) can be easily distinguished after IWS, despite the peak-to-peak distance between them being around 250 nm, which is much below the diffraction limit.

Silver nano-wires are good test samples for estimation of resolution improvement because their dimensions are known. We compared intensity profiles of single isolated NW before and after subtraction, as shown in Fig. 3(a–c). Due to the fact that the diameter of the nanoparticles is much smaller than the illumination wavelength (the diameter of a single wire was 50 nm, while the illumination wavelength was 592 nm), the intensity profile across the wire is given by the line spread function, which is roughly equal to the PSF of the imaging system, consistent with the value obtained from data fitting, which gives 222 nm (Supplementary Fig. S4, black line). We compared this with the image that resulted by subtraction with both a constant α = 0.6 and the IWS method (Supplementary Fig. S4 blue and red lines), and obtained FWHMs of 179 nm and 150 nm, respectively, after fitting. This result confirms that the IWS method allows sub-diffraction resolution improvement up to 33% compared to the confocal image, while avoiding critical over-subtraction distortions.

To demonstrate the potential of the IWS method for biological specimens, we imaged HeLa cells microtubules in both single- and two-photon excitation fluorescence modalities (1PE and 2PE), as well as bovine meniscus collagen fibres by means of second harmonic generation microscopy. The confocal microscopy experiments were performed by Leica TCS STED-CW SP5 microscope, with 100x oil immersion objective NA 1.4, at 592 nm. 2PE and SHG data were obtained from a home-built setup coupled with a Nikon C2 microscope with the following parameters: excitation wavelength 860 nm, focusing objective 100x oil Nikon NA 1.4 (for more details see Methods).

**Figure 4:**
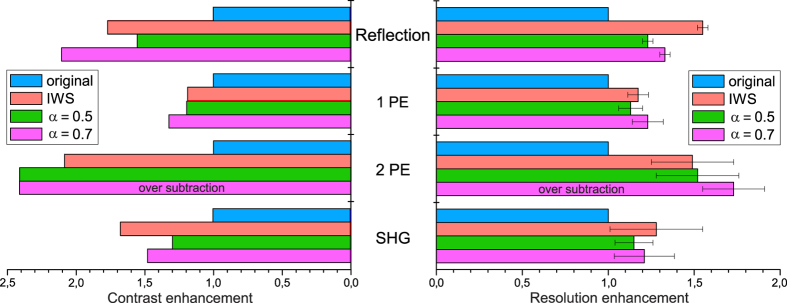
Comparison of contrast (left) and resolution (right) enhancement for different imaging modes: reflection, single-photon excitation fluorescence (1PE), two-photon excitation fluorescence (2PE) and second harmonic generation (SHG); the blue bar represents the original (Gaussian) image, the red is IWS subtraction, and green and magenta are constant subtraction with α = 0.5 and α = 0.7, respectively.

To quantify the contrast enhancement, we performed comparative analysis by calculating the standard deviation (s.d.) of the energy-normalized histograms for each image, as described elsewhere^30^, and normalizing them by the original image data. The obtained values directly reflect the increase of image contrast. Results are summarized in Fig. 4. From the diagram, we can see that in general subtraction microscopy improves the contrast ratio. Overall, the IWS method shows better performance compared with a constant subtraction α = 0.5, with, however, the degree of enhancement depending on the imaging mode. The best results were obtained for the reflection or the non-linear imaging techniques, where the original images have higher noise levels. It should be noted that subtraction with α = 0.7 for two-photon fluorescence introduces severe image distortion, and therefore we excluded this data from the comparison chart (Supplementary Fig. S5). We have chosen a minimum (0.5) and a maximum (0.7) from the recommended range of α coefficients, because for constant subtraction the contrast and resolution enhancements within this interval are linearly proportional to the rescaling coefficient. Therefore the enhancement for α = 0.6 can be easily estimated from the chart as a mean of the presented values. It is important to note, that any subtraction routine also affects the signal-to-noise ratio (SNR) of the images. The exact change in the SNR depends on the imaging mode and data set quality. However, for the presented techniques the IWS method performs well in terms of SNR (for the details, see Supplementary Figs S6 and S7).

In order to quantify the resolution enhancement, we analyzed the intensity profiles (Fig. 3(c,f,i,l)), where the blue and red curves represent the original and the subtracted data respectively. The IWS profiles exhibit better peak-to-peak separation, and small features appear to be more pronounced. By fitting the separate peaks, we estimated the reduction of FWHM and compared the obtained results between different imaging modalities (Fig. 4(b)). The IWS method shows superior performance compared to images subtracted with a constant coefficient set at α = 0.5. Only by increasing the value of α up to 0.7, does the resolution enhancement become comparable for both methods. The total resolution enhancement is similar for all modalities, and shows about 1.3 to 1.5 fold improvement. From calculated PSFs we can estimate the theoretical limits for resolution enhancement in subtraction microscopy, which is 1.66 fold for IWS, and 1.89 or 1.55 for α = 1 and α = 0.5, respectively. As we have shown previously, using a coefficient α = 1 is not practical for crowded samples, because it causes significant image distortion due to over subtraction (see Fig. S2, SI).

Remarkably, the IWS method shows even greater performance for non-linear excitation microscopy, i.e. 2PE fluorescence and SHG, where due to the quadratic nature of the processes the resulting PSFs exhibit narrower peaks. In Supplementary Fig. S8, we compared the numerically simulated subtraction of linear and quadratic PSFs. While constant coefficient subtraction works better in terms of FWHM but much worse in terms of over-subtraction, the IWS method performs better for both parameters. In non-linear cases, IWS method shows an improved resolution and a reduced over-subtraction. This observation is consistent with experimental data, where the IWS subtraction outperforms even α = 0.7 for the SHG mode.

**Figure 5:**
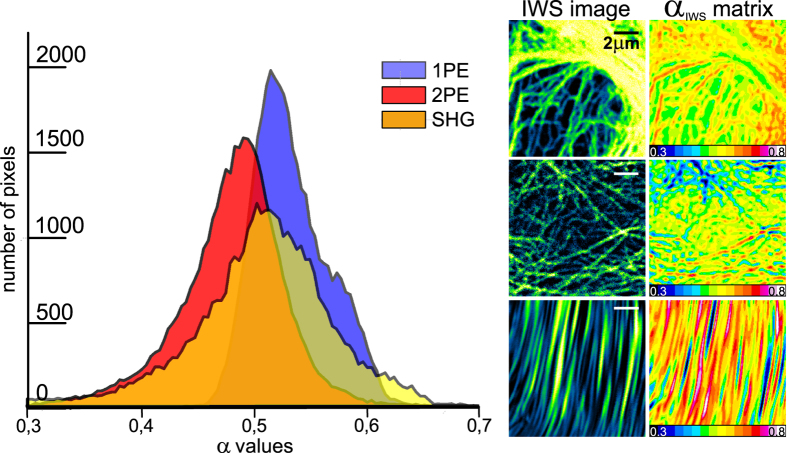
α_IWS_ values distributions for 1PE (blue), 2PE (red) and SHG (yellow) images. On the right are shown the corresponding IWS (1PE, 2PE and SHG from top to bottom) images and 2D plots of α_IWS_ matrices.

It is interesting to analyze the distribution of α_IWS_ values for different images (Fig. 5). From the graph we can see that the coefficient varies over a much broader range compared to recommended constant values. Moreover, the mean of the distributions changes depending on the original image intensity distribution. For instance, the range of α levels in the case of SHG images lies between 0.4 and 0.6 (on the average about 0.52), but the resolution enhancement performance shows even better results compared with constant α equal to 0.7.

**Figure 6:**
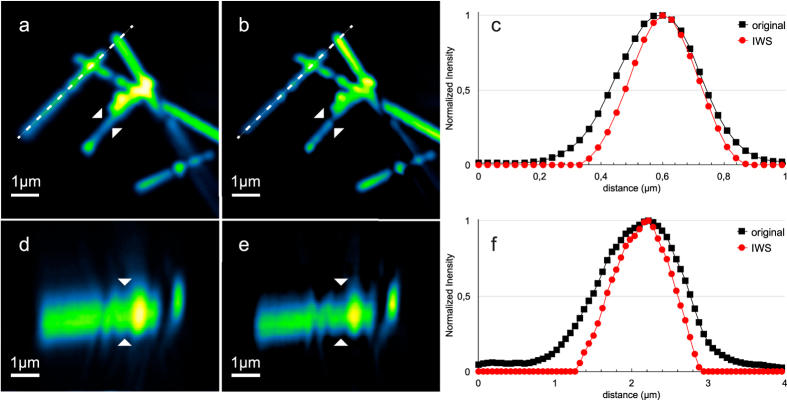
The lateral (**a,b**) and the axial (**d,e**) confocal reflection images of silver nano-wires at 710 nm. (**d**,**e**) images are axial slices along the dashed line indicated in (**a**,**b**) respectively. (**c**,**f**) show the x-y and z intensity profiles along the lines marked with white arrows, respectively. The scale is the same for all directions (x,y,z).

To understand how the IWS method will perform for 3D resolution improvement, we carried out imaging where the doughnut beam is replaced by a bottle beam. Figure 6 shows the resulting confocal reflection images of silver nano-wires illuminated at 710 nm, where (a) and (b) are x-y, and (d) and (e) are x-z projections of original and IWS images, respectively, sectioned along the dashed line depicted in (a) and (b). By fitting the intensity profiles along the lines indicated with white arrows, see Fig. 6(c,f), we were able to obtain 20% resolution improvement for the lateral and axial views. This means that IWS is effective using bottle beam shape too, and does not introduce evident image artefacts. On the contrary, it helps to further enhance both lateral and axial resolutions simultaneously.

The experimental results prove that the proposed IWS approach automatically selects a suitable subtraction coefficient for each pixel, in a way which minimizes over subtraction, and therefore, data loss or distortion. Moreover, it can also be applied to any type of laser scanning microscopy technique, or even for a widefield configuration, such as spinning disk microscopy, where parallelization with multiple beams can be achieved. IWS is especially useful for the label free microscopy modes, such as SHG, where common super resolution methods cannot be utilized.

In this paper we focused our attention on label free and low illumination power applications. Thus an unsaturated doughnut beam was used for the subtraction. However, some authors have proposed the use of a saturated doughnut^21^,^31^. The advantage of such an approach is clear; the central part of the saturated doughnut is significantly narrower compared to unsaturated one. Obviously, this would further improve the resolution enhancement, but at the same time the areas of over-subtraction would also increase. Therefore, applying IWS for subtraction microscopy with a saturated doughnut can also be beneficial for keeping high-resolution improvement while avoiding data loss.

## Conclusion

In this paper for the first time we addressed the over-subtraction issue for subtraction microscopy methods, which limited their use for dense biological samples. As a solution, we proposed a novel approach, where the subtraction coefficient is calculated pixel-by-pixel by taking into account the original image intensity distributions. Our intensity weighted subtraction method significantly reduces the undesired over-subtraction areas, hence avoiding the common problem of image distortions and artefacts. In order to quantify the performance of our method more concretely, we carried out extensive theoretical and experimental studies. We found that the IWS method produces significant contrast enhancement and resolution improvement more than 30% and 20% of its original value in lateral and axial directions, respectively. Further, the method works equally well for both linear and non-linear imaging modes. The latter is particularly important because it indicates that IWS has a great potential for the resolution improvement for techniques like SHG and CARS, where the super resolution methods are unavailable due to the physical nature of the processes. Furthermore, the method is simple, low cost, and yet effective, and can be easily implemented in laboratory conditions.

## Additional Information

**How to cite this article**: Korobchevskaya, K. *et al.* Intensity Weighted Subtraction Microscopy Approach for Image Contrast and Resolution Enhancement. *Sci. Rep.* **6**, 25816; doi: 10.1038/srep25816 (2016).

## Supplementary Material

Supplementary Information
