# Supplementary material for: Intensity Weighted Subtraction Microscopy Approach for Image Contrast and Resolution Enhancement
Source: Sci Rep. 2016 May 12;6:25816. doi: 10.1038/srep25816 (PMC4865876; doi:10.1038/srep25816)
Supplement: Supplementary Information [file srep25816-s1.pdf]

Supplementary Material for

Intensity Weighted Subtraction Microscopy Approach for Image Contrast and Resolution Enhancement

Authors: *Kseniya Korobchevskaya, Chiara Peres, Zhibin Li, Colin J.R Sheppard, Alberto Diaspro and Paolo Bianchini*

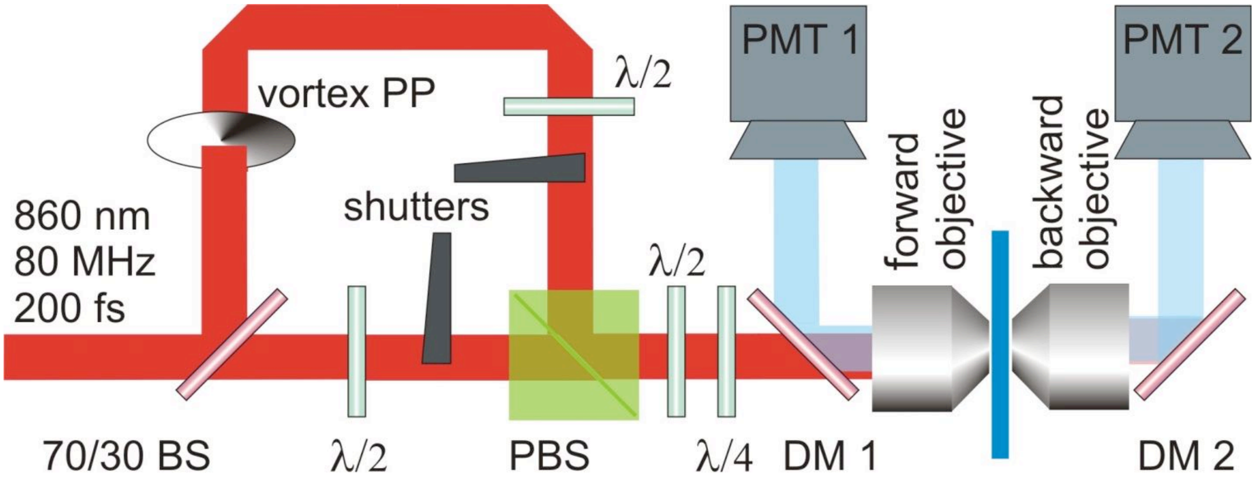

Figure S1. Scheme of experimental setup, where BS is beam splitter, PBS is polarizing beam splitter, vortex PP is vortex phase plate, PMT is photomultiplier tube, DM are dichroic mirror, λ/2 and λ/4 are half- and quarter wave plates, respectively.

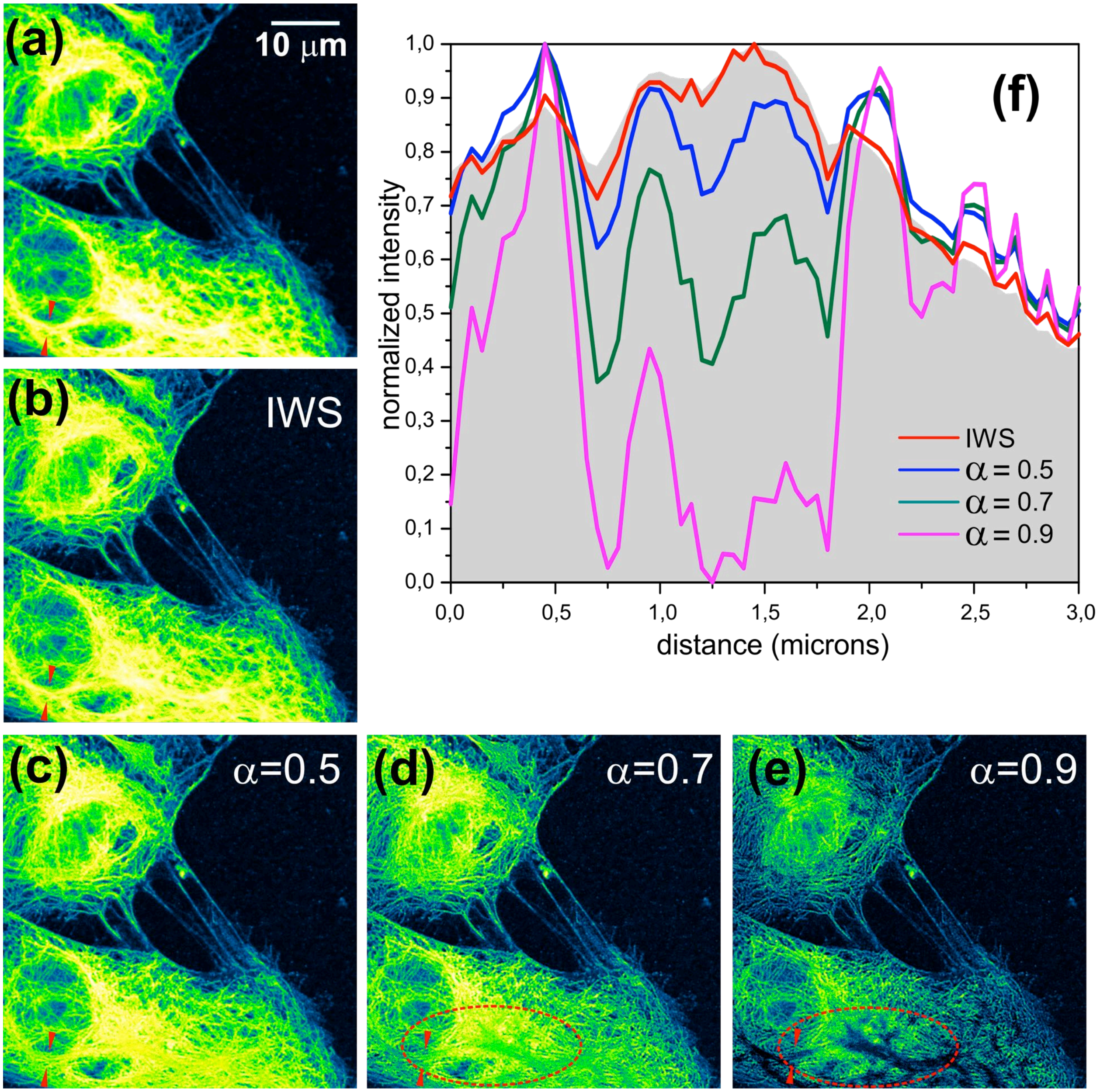

Figure S2. Original (**a**) and subtracted images of HeLa microtubules in 1PE mode excited at 592 nm, where (**b**) is IWS, and (**c-e**) are uniform subtractions with α = 0.5, 0.7 and 0.9, respectively. The over-subtracted area is shown with red ellipse on the figures (**d**) and (**e**). Figure (**f**) shows intensity profile plots along the line indicated with red arrows, where red, blue, green and magenta curves represent profiles for IWS method and α = 0.5, 0.7, 0.9, respectively, while filled grey area shows original image intensity distribution.

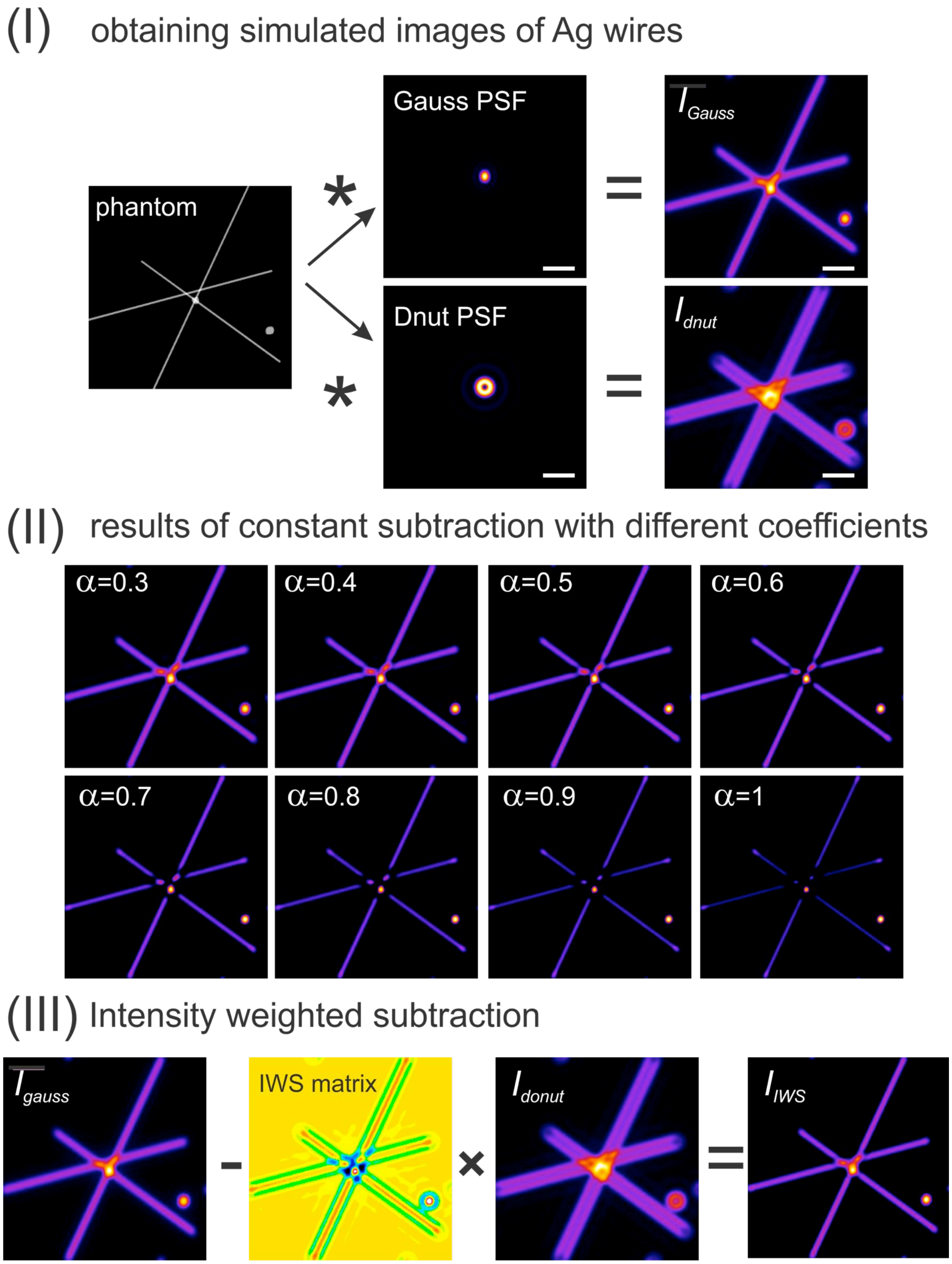

Figure S3. Simulated images of nano wires: (**I**) is phantom image convolved with Gauss and dougnut PSFs (**II**) are results of constant subtraciton with different values of α coefficient, (**III**) IWS mehtod

Fig. S3 shows, step by step, the progress of obtaining simulation images, which are presented in Fig.2 in the manuscript for comparison with experimental data. As we mentioned in the manuscript, the main advantage of the IWS method is minimization of oversubtraction areas. To better appreciate the performance we selected the experimental dataset where three silver nanowires cross each other in a “star” like manner (see Fig. 2). For better comparison with experimental and computational results, we recreated a similar phantom image, which roughly renders the experimental data. From electron microscopy data(data not shown), we know that these silver wires are about 50 nm in diameter and several microns in length, so for the phantom image we draw them accordingly. The resulting phantom image is shown in Fig. S3 (I). As a next step, to simulate Gaussian and dougnut images, we convolved the phantom image with corresponding PSFs, as shown in the figure. The PSFs were obtained following Leutenegger et al. ([28] in the Manuscript) routine, with parameters set similarly to the microscope configuration which we used for experimental measurements. In particular, wavelength was 592 nm, objective NA 1.4, immersion oil refractive index 1.515, polarization was circular. As it could be expected, the calculated images show higher resolution compared with experimental ones, as can be explained by an inmperfect setup aligment, aberrations and slightly different nano-wires dimensions which may vary by up to 30% in width between individual particles. The possibility of particle aggregation also cannot be excluded, because the concentration was selected with the purpose of obtaining densely packed particles in order to investigate the performance of our algorithm for non-sparce samples. Nontheless, the experimental and numerical data give similar results (see Fig.2 in the Manuscript). In particular, the character of the IWS performance in terms of over-subtraction in critical areas (the cross point between the wires) compared with constant subtraction is similar to the experimentaly obtained data.

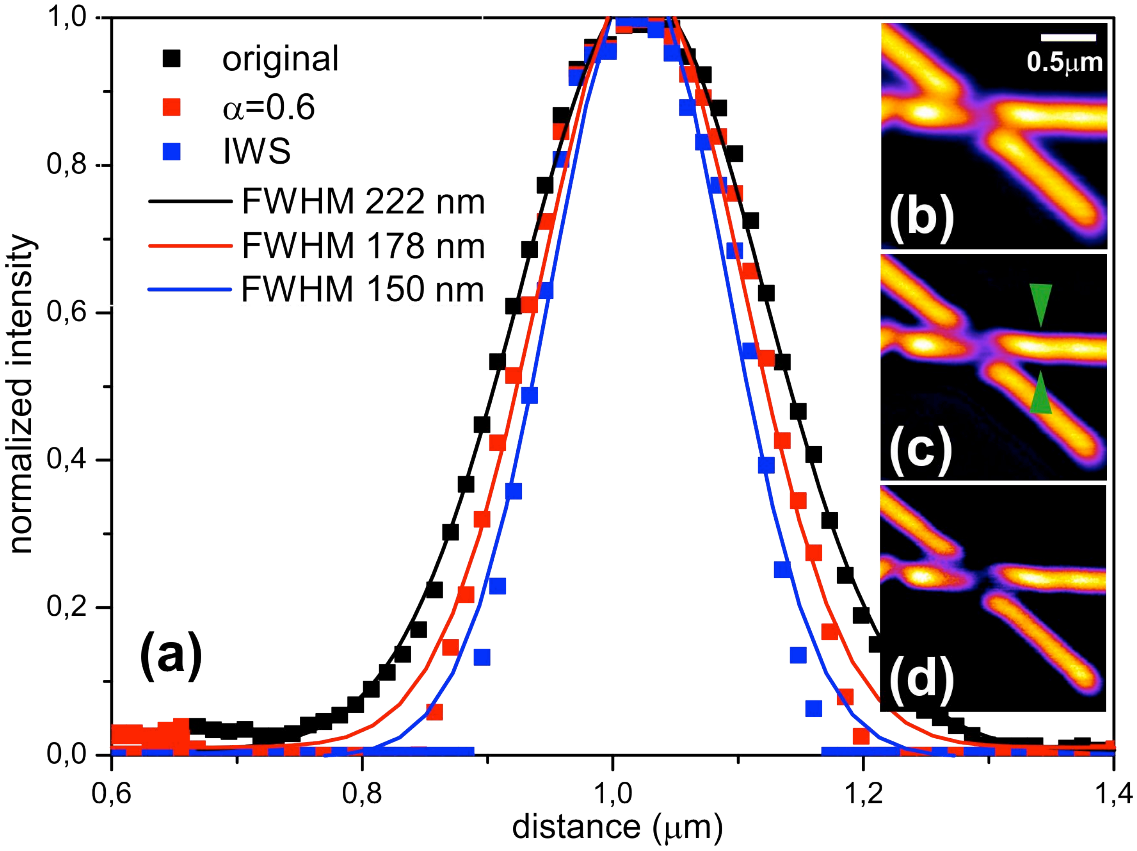

Figure S4. Confocal reflection images of silver NW with their intensity profiles: (**a**) is the profile plots of area indicated with green arrows, where dotted lines are the data plots and solid lines are Gaussian curve fittings; on the inset are shown reflection images (**b**) is Gaussian image, (**c**) is the image after subtraction with α=0.6, and (**d**) is IWS result.

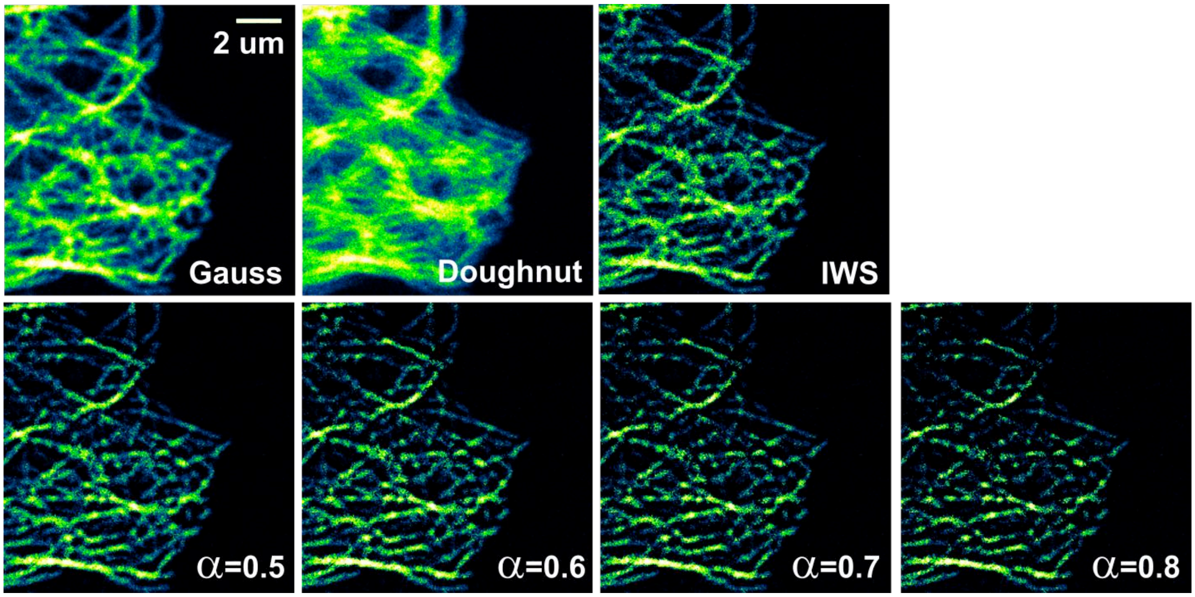

Figure S5. 2PE fluorescence images of HeLa tubulin excited at 860 nm. The upper row demonstrates the Gaussian, doughnut and IWS images respectively, while the bottom row shows the images obtained with constant constant coefficients equal from 0.5 to 0.8, respectively.

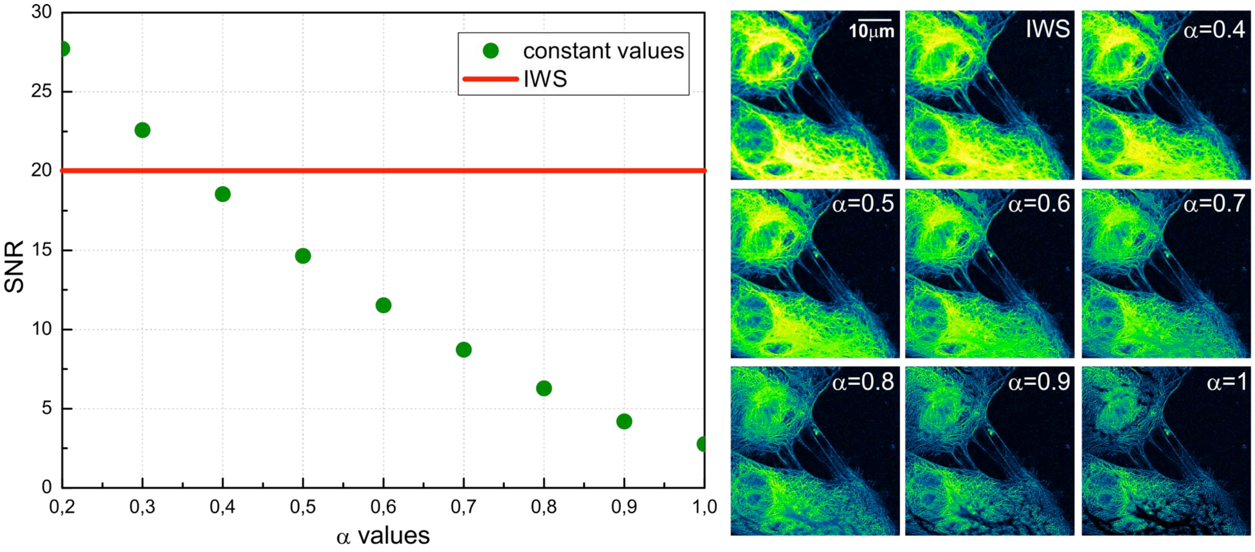

Figure S6. Graph at the left shows the comparison of SNR change after constant subtraction with different alpha values and IWS. At the right corresponding images are shown in following order: original, IWS, and alpha from 0.4 to 1, respectively.

To estimate the change in signal-to-noise ration (SNR) after subtraction we analyzeed our data with ImageJ plugin developed by Daniel Sage at the Biomedical Image Group, EPFL, Switzerland [1]. The algorithm compares original and processed images, and calculates the SNR (Signal-to-noise ratio, expressed in dB), PSNR (Peak signal-to-noise ratio, expressed in dB), RMSE (Root mean square error), and MAE (Mean absolute error) according to definitions by Gonzalez et al. [2]. The table 1 presents the values obtained for the dataset used for Fig. S2 and Fig. S6.

Table 1.

Subtraction type	SNR	PSNR	RMSE	MAE
IWS	20.0	27.6	10.6	7.8
α=0.4	27.7	35.3	4.4	2.8
α=0.5	22.6	30.2	7.9	4.8
α=0.6	18.5	26.1	12.6	7.4
α=0.7	14.7	22.2	19.8	11.6
α=0.8	11.5	19.1	28.3	16.6
α=0.9	8.7	16.3	39.1	23.3

As it could be expected, the subtraction decreases the SNR ratio. However, the exact result is dependend on imaging mode and characteristics of data set. The summary of SNR change for different imaging techniques is shown in Fig. S7.

In the column chart below are shown the comparison data of SNR for different imaging techniques and for most common subtraction coefficients (0.5 and 0.6) vs our intensity weighted subtraction method. As can be seen from the Fig. S7, IWS method performs better for one- and two-photon fluorescence images, and slightly worse for reflection and SHG. On average, the IWS SNR enhancement is comparable or superior to the constant subtraction.

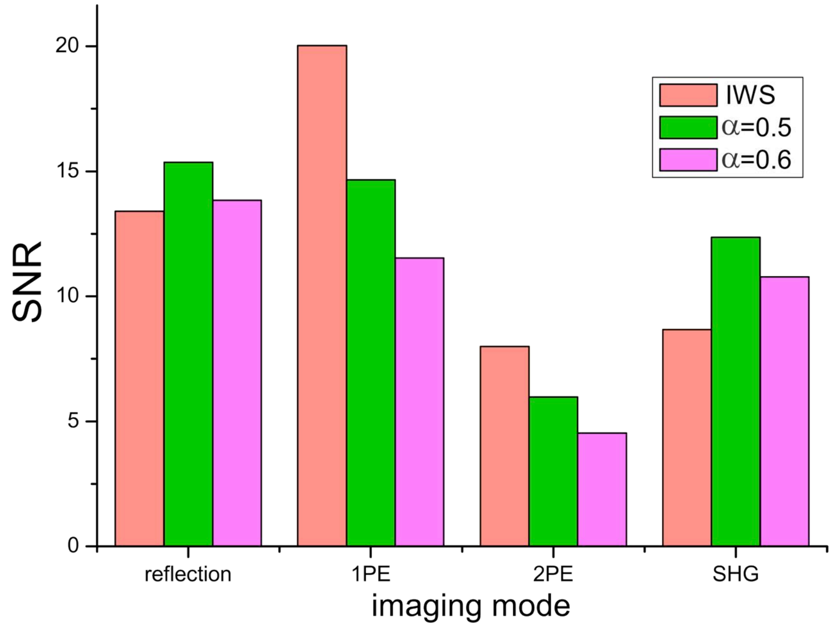

Figure S7. Effect of constant (α=0.5 and α=0.6) and IWS subtraction on SNR for different imaging modes: reflection, one-photon excitation, two-photon excitation and second harmonic generation. The data was obtained after analysing images shown in Fig.3 in the Manuscript.

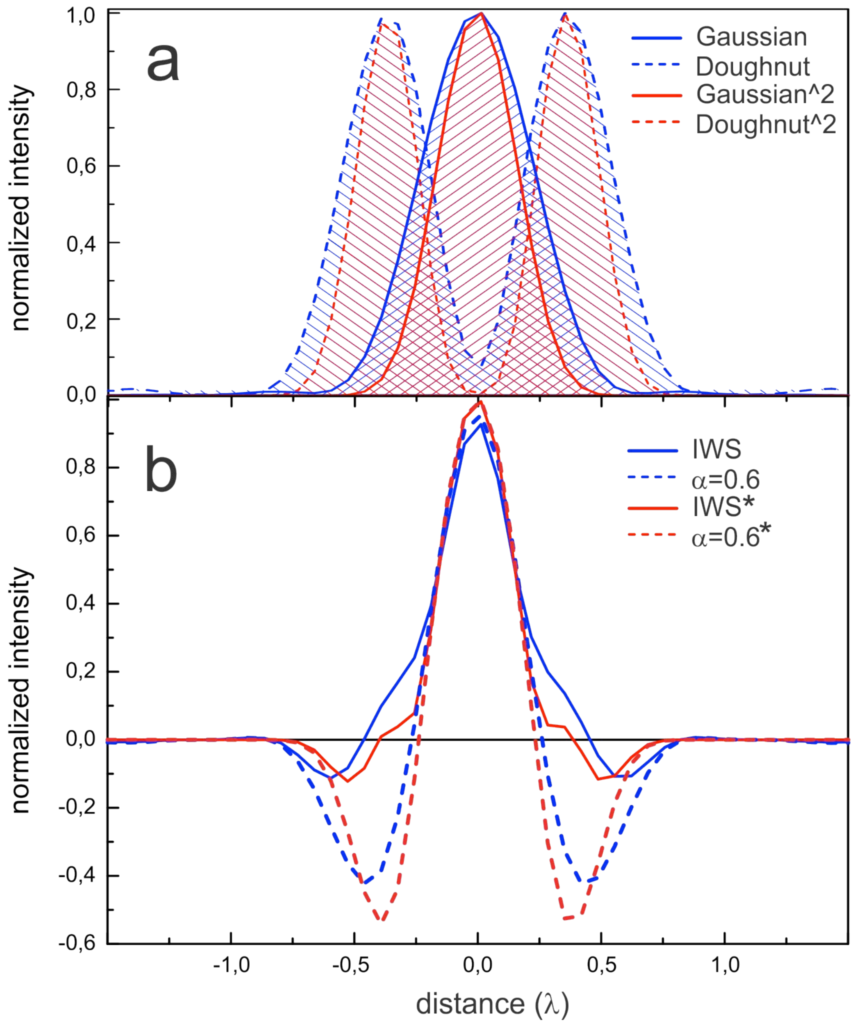

Figure S8. (**a**) shows simulated intensity profiles for linear (blue) and quadratic (red) excitation PSFs, where solid outlines show Gaussian and dashed doughnut PSFs, respectively; (**b**) shows resulted PSFs after constant subtraction with α=0.6 (dashed lines) and IWS (solid lines), where blue lines represent results for linear, and red lines for quadratic excitations (marked with *), respectively.

References

1. http://bigwww.epfl.ch/sage/soft/snr/
2. Gonzalez RC, Woods RE. Digital Image Processing. 3rd ed. Upper Saddle River: Prentice Hall; 2008.
